# Development of an eHealth-enhanced model of care for the monitoring and management of immune-related adverse events in patients treated with immune checkpoint inhibitors

**DOI:** 10.1007/s00520-023-07934-w

**Published:** 2023-07-22

**Authors:** André Manuel da Silva Lopes, Sara Colomer-Lahiguera, Célia Darnac, Stellio Giacomini, Sébastien Bugeia, Garance Gutknecht, Gilliosa Spurrier-Bernard, Veronica Aedo-Lopez, Nuria Mederos, Sofiya Latifyan, Alfredo Addedo, Olivier Michielin, Manuela Eicher

**Affiliations:** 1 Institute for Higher Education and Research in Healthcare (IFS), Faculty of Biology and Medicine, University of Lausanne (UNIL), Lausanne University Hospital (CHUV), Route de la Corniche 10, CH-1010 Lausanne, Switzerland; 2 Department of Oncology, Lausanne University Hospital (CHUV), Rue du Bugnon 46, CH-1011 Lausanne, Switzerland; 3 Department of Oncology, Geneva University Hospital (HUG), Rue Gabrielle-Perret-Gentil 4, 1211 Genève, Switzerland; 4 MelanomeFrance, Teilhet, France; 5 Peter MacCallum Cancer Centre, Melbourne, Victoria Australia; 6 Medicine and Dentistry, Health Sciences, University of Melbourne, Melbourne, Victoria Australia

**Keywords:** Patient-reported outcomes, Model of care, Immune-related adverse events, Remote symptom management, Self-management support

## Abstract

**Purpose:**

The use of electronic patient-reported outcome (ePRO) data in routine care has been tied to direct patient benefits such as improved quality of care and symptom control and even overall survival. The modes of action behind such benefits are seldom described in detail. Here, we describe the development of a model of care leveraging ePRO data to monitor and manage symptoms of patients treated with immune checkpoint inhibitors.

**Methods:**

Development was split into four stages: (1) identification of an underlying theoretical framework, (2) the selection of an ePRO measure (ePROM), (3) the adaptation of an electronic application to collect ePRO data, and (4) the description of an ePRO-oriented workflow. The model of care is currently evaluated in a bicentric longitudinal randomized controlled phase II trial, the IePRO study.

**Results:**

The IePRO model of care is grounded in the eHealth Enhanced Chronic Care Model. Patients are prompted to report symptoms using an electronic mobile application. Triage nurses are alerted, review the reported symptoms, and contact patients in case of a new or worsening symptom. Nurses use the UKONS 24-hour telephone triage tool to issue patient management recommendations to the oncology team. Adapted care coordinating procedures facilitate team collaboration and provide patients with timely feedback.

**Conclusion:**

This report clarifies how components of care are created and modified to leverage ePRO to enhance care. The model describes a workflow that enables care teams to be proactive and provide patients with timely, multidisciplinary support to manage symptoms.

**Supplementary Information:**

The online version contains supplementary material available at 10.1007/s00520-023-07934-w.

## Introduction

Immune checkpoint inhibitors (ICI) have become part of the standard of treatment for an expanding range of cancer types [1]. Despite having shown a lower toxicity profile compared to other treatments, immune-related adverse events (IrAE) caused by ICI can nevertheless be severe and potentially fatal [2, 3]. The likelihood of experiencing an IrAE is influenced by treatment modality: between 40 and 75% of patients treated with a single ICI experience an IrAE (any grade), with 10 to 30% experiencing severe events (grade ≥ 3) [3, 4]. About 95% of patients experience at least one IrAE when treated with combined ICI, and nearly 60% of patients experience at least one severe IrAE [5].

These IrAE are notably heterogeneous, occasionally resembling disease progression and mimicking auto-immune conditions [4]. Severe IrAE can be persistent or occur several months into and beyond treatment [6–8], thus adding on to the already considerable acute and chronic symptom burden patients experience.

Patient education and symptom self-management, particularly self-monitoring, contribute to more timely detection of IrAE, better short-term outcomes for patients, and lower incidence of chronic symptoms [9, 10]. However, patients treated with ICI may not be sufficiently supported in that domain [11, 12]. Mild symptoms are often under-recognized and under-reported by patients and clinicians, though they may be indicative of more serious developing conditions impacting quality of life [13, 14]. Close and frequent communication between patients and healthcare providers is thus essential in preventing severe IrAE. Information flyers and telephone follow-up targeting symptoms related to ICI treatment have been used to support patients and anticipate the delivery of care [15]. However, evidence-based procedures to monitor and manage them in a real-world setting are still lacking [16, 17].

The use of patient-reported outcome measures (PROM) has been shown to improve symptom detection, monitoring, and management by empowering patients to convey their perception of symptoms to healthcare providers, while also providing valuable treatment safety and tolerability data [12, 13, 18, 19]. Electronic PROM (ePROM) can play a role in shared clinical decision support by influencing treatment decisions and improving the scope and efficiency of patient-provider communication [20–22]. Remote real-time symptom reporting and monitoring facilitated by the use of ePROM may lead to more accurate insights into patients’ health status than delayed self-reports [23].

Studies involving the use of electronic PRO (ePRO) data in oncology reported a decrease in hospitalization rates and emergency department visits, with favorable outcomes on quality of life, perceived self-efficacy, and overall survival [13, 24]. How these studies’ interventions mobilized and interacted with existing care structures and procedures to produce beneficial outcomes is seldom described in detail [25]. Some interventions used ePRO to assess symptoms remotely as complementary clinical decision support to modify treatment or to refer patients to emergency or acute care services, among others [13]. To our knowledge, no studies targeting the remote management of symptoms of patients treated with ICI have detailed the conception and integration of ePRO-based care models, within existing care delivery structures.

In this report, we describe the development of a model of care that leverages ePRO data to monitor and manage symptoms in patients treated with ICI, in an outpatient care setting. This model is currently being tested in a randomized controlled phase II trial, the IePRO trial, at two Swiss university hospitals (ClinicalTrials.gov Identifier: NCT05530187).

## Toward the development of an ePRO-based model of care

Development of the IePRO model of care took place between November 2020 and November 2021. A team of four physicians and five nurses of the participating institutions’ oncology departments and one patient-representative collaborated in the creation of its core components and their integration in the existing workflows of each hospital. All members had previous experience in collecting and interpreting PRO data in clinical oncology trials. Two nurses have published research on PROM aimed toward patients treated with ICI [26]. The patient representative was identified by screening Swiss and French patient advocacy groups related to oncology. A brief in-person interview allowed to assess their knowledge of ICI and their side effects, expertise in using PROs, and experience in collaborating in clinical trials.

This ePRO-based model of care was developed in four stages: (1) identification of an underlying theoretical framework, (2) selection of an ePROM, (3) adaptation of an electronic mobile application to collect ePRO data, and (4) ePRO-oriented workflow and clinical roles.

### Theoretical framework

As ICI-related symptoms may add to the symptom burden of patients, effective management of these symptoms requires a holistic approach. To reflect upon and address the complexity and resources required for symptom management, we grounded the development of this intervention in the eHealth Enhanced Chronic Care Model (eCCM) [27], which is itself an extension of the Chronic Care Model (CCM) [28, 29].

The major components of the CCM, community resources and health systems, are complemented by eCommunity and eHealth in the eCCM [27]. eHealth includes the digital tools and resources available to patients that complement those provided by the healthcare system. Online communities and health-related social networks constitute the eCommunity, which supports patient engagement and activation for self-management.

**Fig. 1:**
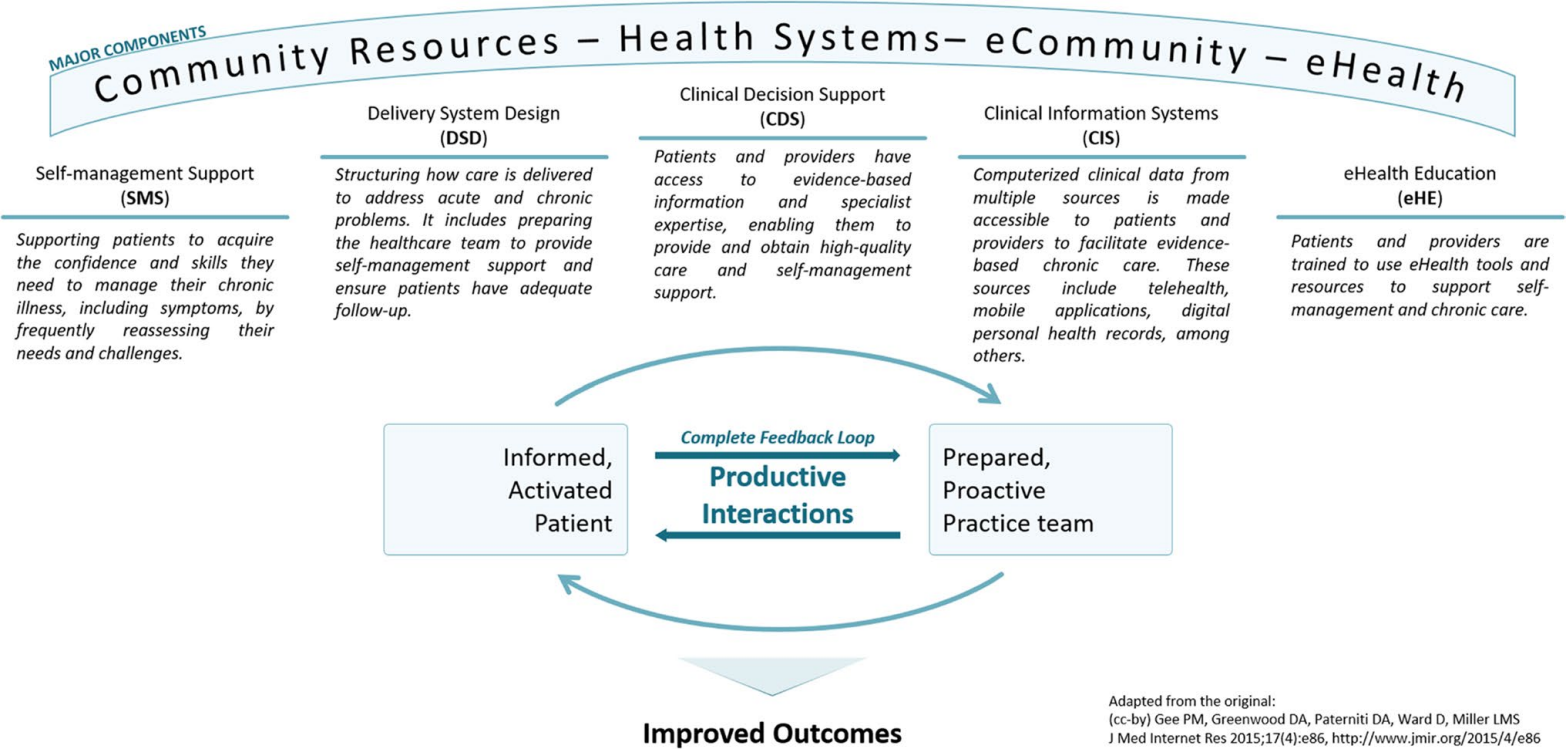
The eHealth Enhanced Chronic Care Model (eCCM), adapted from: Gee PM, Greenwood DA, Paterniti DA, Ward D, Miller LMS. The eHealth Enhanced Chronic Care Model: A Theory Derivation Approach. Journal of Medical Internet Research 2015;17:e86. 10.2196/jmir.4067. The original is licensed under a Creative Commons Attribution license (CC-BY)

The major components of the eCCM encapsulate five smaller interdependent components: self-management support (SMS), clinical decision support (CDS), delivery system design (DSD), clinical information systems (CIS), and eHealth education (eHE). These are brought together to ensure informed and activated patients interact with prepared and proactive practice teams, leading to satisfying encounters and improved outcomes [27]. They are described in further detail in Fig. 1.

We address each of these smaller components and clarify their role in achieving productive interactions between patients and care providers as we describe the following development phases of the IePRO model.

### Selection of an ePROM

Active discussions between the model development team allowed to identify an ePROM of particular interest, to both clinicians and patients. The patient-representative mobilized her patient-advocacy network to collect and convey general perceptions on existing PROM, such as their perceived advantages and disadvantages to assess symptomatic ICI-related toxicity, via e-mail. The PRO version of the Common Terminology Criteria for Adverse Events (PRO-CTCAE™) item library was considered comprehensive and suitably flexible, measuring a broad spectrum of symptoms [26, 30]. Using the results of a previous Delphi study, we identified a set of 37 priority PRO-CTCAE™ items for routine symptom monitoring in this patient population, which compose the IePRO trial’s weekly symptom questionnaire [31].

Patients participate in the IePRO trial for the first six months of their ICI treatment. Because the majority of IrAE occur within the first three to four months of treatment [7], active symptoms are re-assessed daily for the first three months, using a modified recall period of 24 hours, between weekly questionnaires. In addition, patients can add any of the 80 PRO-CTCAE™ items to the daily and weekly assessments.

### Adaptation of an electronic mobile application

The main goal in using an ePRO application is to enhance self-management support (SMS). As an eCCM component, SMS includes the provision of tools and resources for patients to acquire the skills and confidence to manage and monitor their health condition [32]. We adapted an application developed by Kaiku Health Ltd., where the developed ePROM was integrated. Studies using similar iteration versions of the Kaiku Health app have reported high agreement across patients and providers on its ease of use and high levels of satisfaction and relevance for clinical practice [33, 34].

**Fig. 2:**
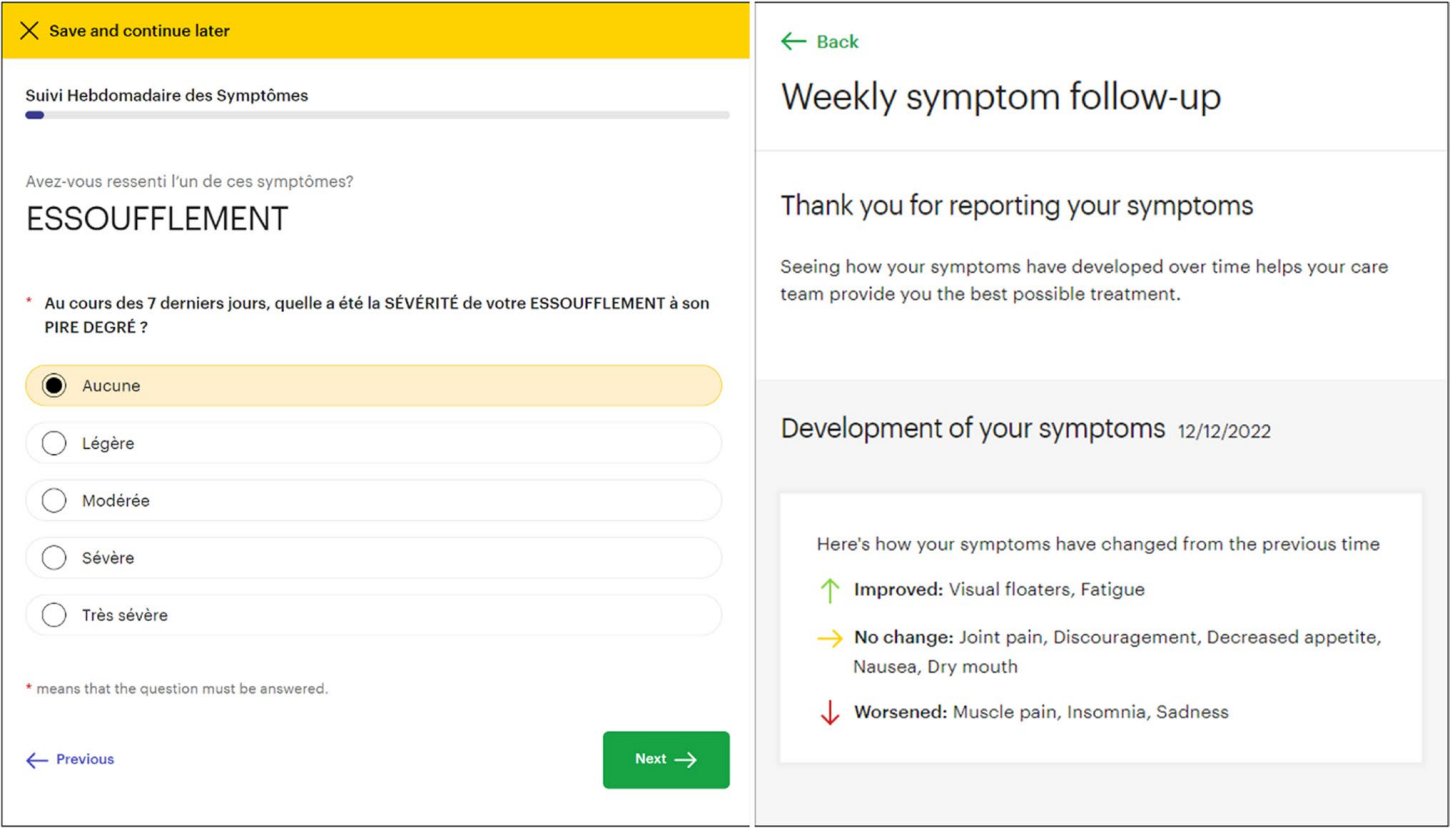
ePRO application—questionnaire interface (left) and patient feedback view (right)

The application sends patient reminders to fill out the ePROM at the previously mentioned time points, to facilitate data collection [35]. It displays all previous replies to any questionnaire, facilitating self-care and self-monitoring tasks [35]. In addition, at the end of each symptom questionnaire, a summary portraying symptom evolution is displayed (Fig. 2).

Since these features may increase symptom awareness, guidance to perceive their detection as empowering to manage and prevent complications is required, as they can also be perceived as signs of deterioration or disease progression, decreasing perceived self-efficacy [36].

To enable patients to navigate the complete item bank of the PRO-CTCAE™, a symptom selection screen was developed in collaboration with patients from the oncology department and the patient-representative, through a card-sorting exercise. Results were used to adapt the screen presented to patients allowing adding symptoms to be monitored.

Integration of ePRO data into clinical information systems (CIS) like the patient’s electronic health record (EHR) is a desired outcome, as it can decrease the technological burden and enhance accessibility of data [13, 32, 37]. The IePRO trial is conducted in two university hospitals operating different EHR platforms. An initial assessment for readiness to implement PRO data concluded that the CIS could not be modified to directly integrate ePRO data in similar ways. Nurses are thus prompted to access the application directly via e-mail when patients report new symptoms.

### Development of an ePRO-oriented workflow and clinical roles

In the eCCM, delivery system design (DSD) relates to how care is coordinated and delivered across the network of health resources. The participating oncology departments treat a similar range of tumor types and number of patients, with similar provider team compositions. Physicians and nurses involved in direct patient care revealed service-level and provider-level barriers such as the time required to navigate, collect, and process PRO data, the integration or lack thereof within the EHR, and internal communication pathways to ensure the continuity of care [38, 39]. These barriers were included in the development of the model of care.

**Fig. 3:**
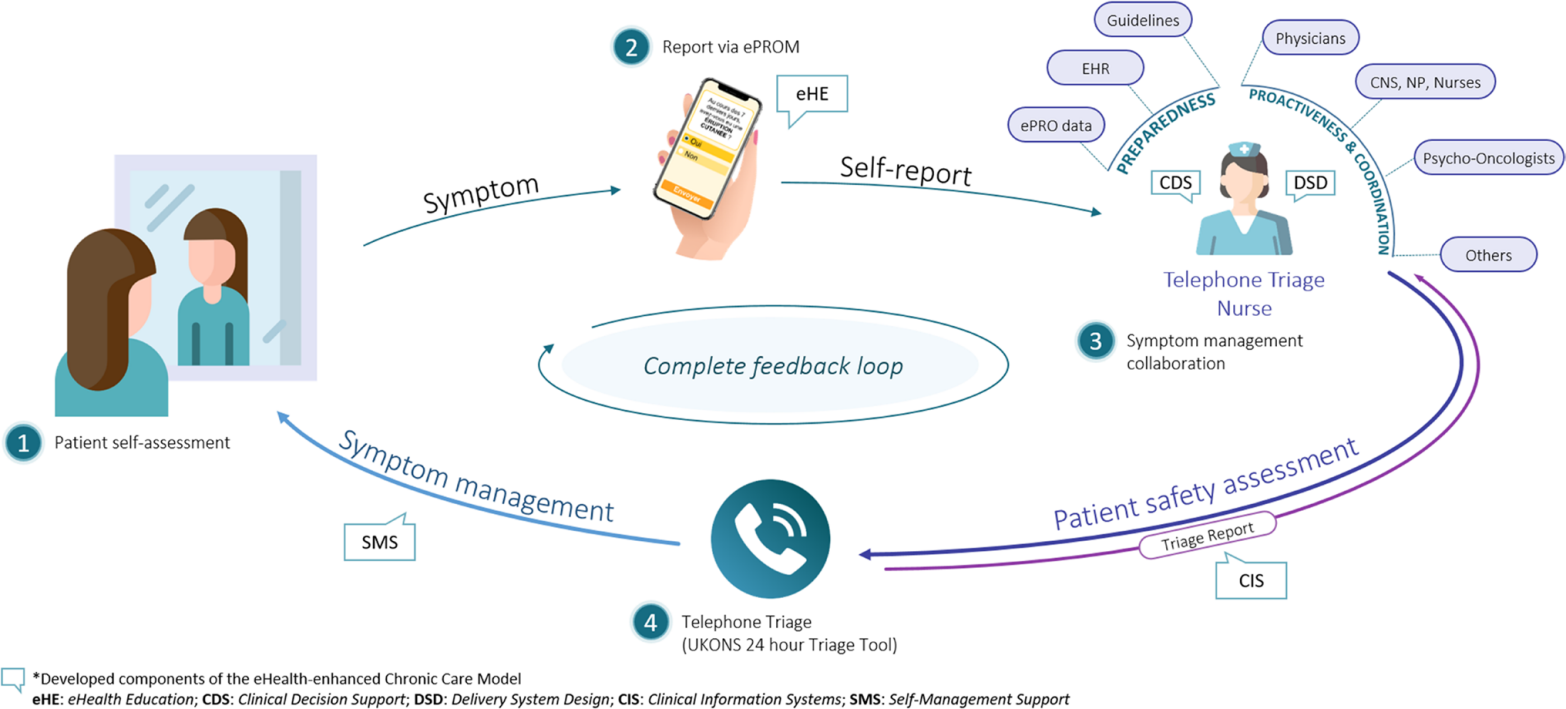
Overview of the IePRO model of care: patients perform self-assessment (1) and declare potential symptoms using the symptom ePROM in the electronic application (2). Telephone triage nurses review PRO data and coordinate with the oncology team preemptively when necessary (3) and contact patients by telephone using a standardized triage process (4)

We hereafter describe how patients are engaged, the triage process and the triage nurse role, and the nurse-physician coordination to provide care. An overview of the model is featured in Fig. 3, and components of the eCCM represented in the IePRO model are summarized in Table 1.

**Table 1:** Components of the eCCM in the IePRO model of care

Components of the eCCM	Related element of the IePRO model of care	Description
Self-management support (SMS)	Electronic mobile application	The electronic mobile application containing the ePROM: • Communicates patient-reported data in real time • Engages patients with automated reminders to encourage self-assessment of symptoms • Provides patients with a chart portraying the evolution of their symptoms over time
	Telephone triage process	SMS is provided to patients by the triage nurses using the French translation of the UKONS 24-hour triage tool and available internal and international guidelines in symptom and IrAE management.
Delivery system design (DSD)	Redesigned care coordination	• Triage nurses call patients in the event of a new or worsening symptom and administer self-care and self-management support via telephone call. • Symptoms are relayed to the oncology care team via e-mail or telephone call, according to UKONS 24-hour triage tool recommendations. • The triage process is documented in the patient’s EHR. • Follow-up measures put in place are communicated with the broader team using e-mail.
Clinical decision support (CDS)	Telephone triage algorithm	The UKONS 24-hour triage tool algorithm outputs actionable recommendations for self-care and self-monitoring of symptoms, with clear clinical management guidance including if an in-person assessment is recommended.
	Internal symptom management guidelines	Both sites have internal evidence-based symptom management guidelines, based on international guidelines that support clinicians when reviewing the recommendations issued from the triage algorithm.
Clinical information systems (CIS)	Telephone triage report	EHR notes are standardized according to the contents of the triage log form of the UKONS 24-hour triage tool, enabling access to triage reports by all healthcare providers.
eHealth education (eHE)	Patient eHealth education	Patients are guided in the use of the electronic application and the extent of its functionality. Introductory information flyer is provided, and further education is provided in-person or over the phone by nurses.
	Provider eHealth education	Providers were trained in the use of the UKONS 24-hour telephone triage tool and on the use of the ePROM application to monitor patient-reported symptoms.

#### Patient engagement

As in the eCCM, informed and activated patients are key to create productive interactions with the healthcare providers [27]. Patients receive information on treatment side effects from clinical nurse specialists (CNS), physicians, and nurses. Triage nurses present the electronic application to the patient, provide a setup guide (online supplement A), and assist in its configuration.

Patients fill out the 37-item ePROM within the first week of ICI treatment by logging in to the online or mobile (smartphone) version of the application. They are prompted to complete subsequent daily and/or weekly questionnaires via an e-mail reminder or push notifications. Patients are made aware their answers in the ePROM will be reviewed by a team of triage nurses on weekdays between 8 and 12 pm. As part of the standard of care, patients are nevertheless encouraged to contact their oncology team directly in case any of any symptoms self-perceived as a cause of immediate concern.

#### Telephone triage nurses and triage process

Telephone triage nurses are the main vector of communication between the patient and the clinical oncology team in the IePRO model. This role was developed and reviewed with oncology physicians, nurses, and CNS. For some oncology subspecialties, the CNS provide sporadic telephone consultations for the most vulnerable patients; therefore, clarifying the role of triage nurses was essential to avoid confusion among providers and patients. While triage nurses work as gatekeepers, helping patients access and appropriate level of care, CNS are a resource to ensure evidence-based symptom management and provide highly specialized care.

Triage nurses were trained to use the United Kingdom Oncology Nursing Society (UKONS) 24-hour triage tool [40]. It was translated and validated in French, in collaboration with the UKONS, for use in the IePRO trial. Two members of the nursing team in one hospital received online training directly from UKONS, who trained the remaining three nurses.

The tool standardizes remote symptom assessment and provides clear guidance on remote symptom management. Triage procedures are triggered when triage nurses detect a new or worsening symptom in the ePRO application. The triage algorithm outputs three types of alerts according to symptom severity: (1) green alerts are issued for mild and stable symptoms where self-management support is recommended, (2) amber alerts represent symptoms that may increase or decrease in severity and thus require a new assessment within 24 hours, and (3) red alerts are issued when symptoms are moderate-to-severe, and in-person assessment is recommended. Nurses log triage procedures in the EHR using an electronic version of the tool’s triage log form. Since CIS integration was not possible, triage nurses alert physicians, CNS, and nurse practitioners of triaged symptoms and of their recommendations by sending a daily summary of all calls.

In the event of a green alert, triage nurses provide self-care guidance, and the oncology team is notified via the e-mail summary. When an amber alert is issued, the oncology team is immediately contacted via e-mail to validate the triage nurses’ assessment and determine if any additional care should be provided. More than one amber alert or at least one red alert triggers triage nurses to call the patient’s oncology physician to seek their specific recommendations and call the patient back to convey the latter. As this model of care is complementary to the standard of care, outside of the triage nurses’ operating schedule, standard procedures apply.

**Fig. 4:**
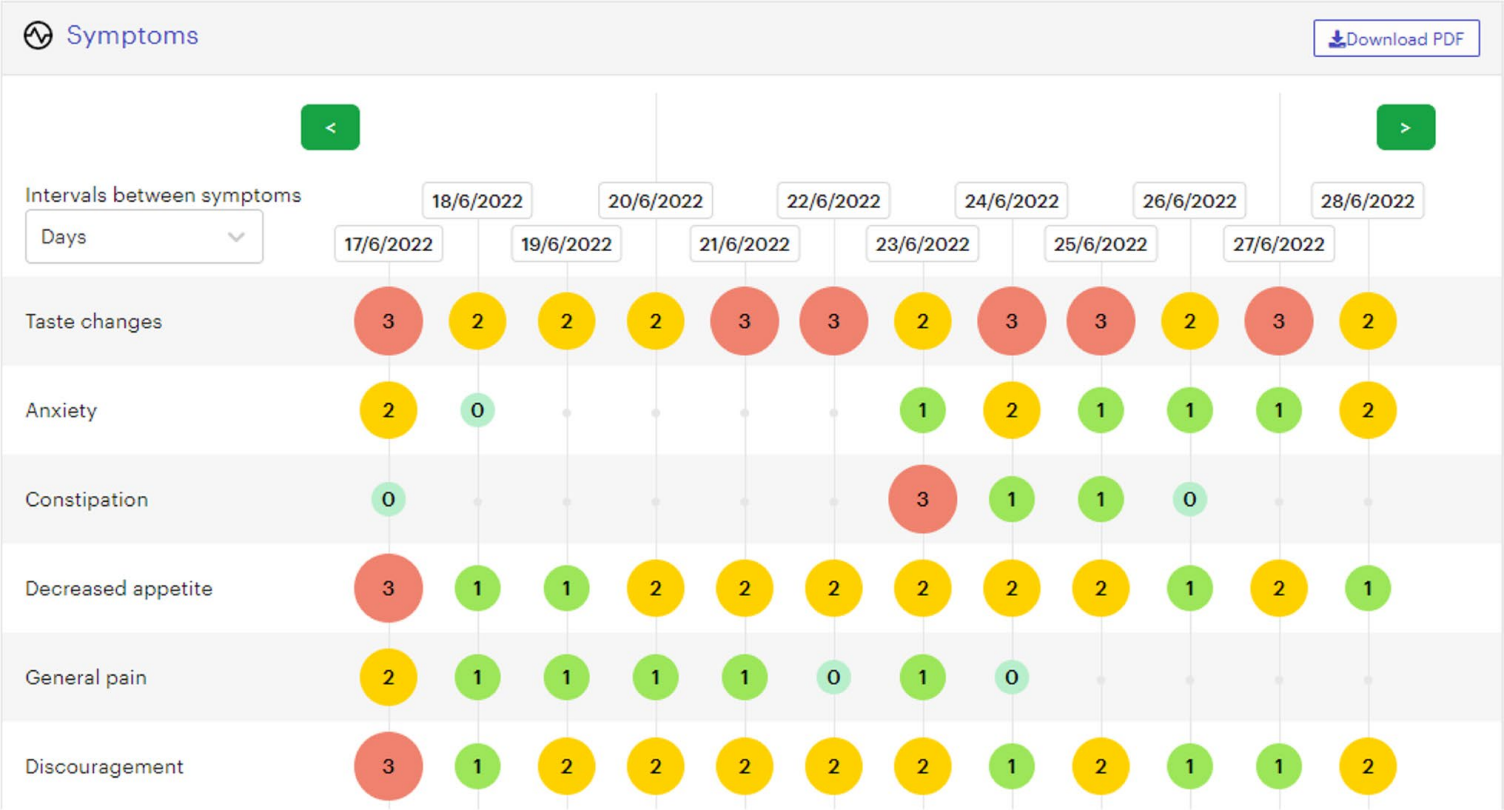
ePRO app—triage nurse’s view

As part of the eHealth education (eHE) component in the IePRO model, triage nurses were trained extensively with the ePROM application between April and November 2021. It presents nurses with a visual and numerical representation of the reported symptoms (Fig. 4) that reflect a combination of PRO-CTCAE™ attributes (frequency, severity, interference, amount, and presence/absence). As outlined in the eCCM, access to this type of remote patient-reported symptom data enables the care team to be proactive and prepared for triage calls in advance [27].

#### Role of physicians and other healthcare professionals

Physicians are the primary collaborators with the triage nurses and are responsible for reviewing triage reports. When their assessment differs from the nurse’s, the physician is to contact them and the patient to provide their recommendation. Triage nurses and physicians may also forward requests to other professionals, such as psycho-oncologists and physiotherapists. An automated e-mail reminder to follow-up on and assess previously reported symptoms is sent in the morning of each in-person patient visit.

### Assessing usability of the ePRO application and acceptability of the model of care

Assessment of the usability of the ePRO application and the acceptability of the model of care from the patient’s perspective takes place up to two weeks after study discontinuation. The mobile application’s usability and the model of care’s acceptability are assessed through semi-structured interviews with patients. Based on the mHealth app usability questionnaire (MAUQ) by Zhou et al. [41], interview guides have been developed. Items were grouped by their scope and nine open-ended questions were formulated by the research team, available as an online supplement (Supplement B).

A semi-structured patient interview guide to assess the acceptability of the model of care was created by the research team, using the definition of acceptability by Sekhon et al. [42]. Questions were derived from the seven constructs of acceptability: “affective attitude,” “burden,” “ethicality,” “intervention coherence,” “opportunity costs,” “perceived effectiveness,” and “self-efficacy” (Supplement C).

To assess the intervention’s acceptability from the healthcare provider’s perspective, an interview guide was developed based on the Consolidated Framework for Implementation Research (CFIR) [43]. It includes questions addressing the model of care’s characteristics, the outer and inner settings, the characteristics of the individuals using the model, and the process of implementation (Supplement D). Acceptability of the model of care will be assessed up to two weeks after the end of the trial.

## Discussion

The IePRO model of care supports the detection and timely management of symptoms of patients treated with ICI. It represents a pragmatic research approach to the use of ePRO data in the context of two university hospitals that retain minor differences in resources and infrastructure, standard operating procedures, and care culture. It describes workflow changes that exist in parallel to usual care, complementing clinical activity and outlining a closed feedback loop between patients and care providers based on electronic monitoring of PRO data.

We consider this model of care to have notable strengths. Due to the potential of symptomatic IrAEs to become chronic conditions, there is a need for forward-looking transformations in care delivery that focus on both short and long-term care [8]. The model ensures that pre-existing and new symptoms are equally taken into account and that the full range of resources is mobilized to manage them. Contrasting with other trials using PRO-CTCAE™ items [44, 45], it accommodates the use of the full item library, lending itself to the heterogeneous toxicity of ICI. Alternating weekly fixed-length and adaptive daily questionnaires enables the detection of quick and sudden fluctuations in symptom severity, while potentially minimizing patient burden. Guided by the eCCM, the model aligns with recommendations from previous studies and with recent guidelines for implementing PRO in routine care, despite preceding them [13, 25, 46, 47]. As part of a clinical trial, some of the eCCM’s components were not developed in this iteration, namely, the community and eCommunity. Integration of these components in the future should be considered to broaden the support for patient self-management.

The model ensures patients receive tailored feedback every weekday they complete a questionnaire, without the requirement of a hospital visit. This closed feedback loop attempts to value the time patients invest in symptom reporting and encourage patients to continue self-monitoring.

Some challenges relating to future implementation, patient engagement, the triage nurse role, and the clinical and technological burden remain. There are no CNS and nurse practitioners available in one of the sites, and thus, the triage nurses are likely to more often strictly rely on physician collaboration to manage symptoms. In the same site, physician teams are less differentiated across tumor types, which may simplify the flow of information with nurses. E-mail reports for mild to moderate symptoms may not facilitate as timely of an intervention as direct telephone or face-to-face contact. However, the care teams agreed it would be the most effective way to request multidisciplinary support and update all relevant parties on patient status. This may increase the burden on triage nurses to obtain a timely reply. An integrated system in the EHR could potentially save time and provide a clearer transfer of responsibility across the oncology team.

The allocation of dedicated resources is recommended for successful implementation of PRO data in routine care, as there is the possibility of increased clinical burden [21, 24, 48]. Training in interpreting PRO data was focused on triage nurses, as time and technical constraints prevented deeper integration with the broader oncology team. Universal access to ePRO data would decrease friction, despite being more resource-intensive in its initial deployment [48]. Currently, triage nurses require more time to process data and create an accessible output for the oncology team. There is a clear risk of incomplete or inaccurate information between the triage reports and the self-reported patient data, which constitutes the most significant limitations of this model. Our preliminary experiences in the IePRO trial suggest clear benefits in training all providers to use PRO data and in integrating it directly in the EHR to minimize the technological burden. Weekly meetings between the nursing triage staff and the PIs of the IePRO trial, who are involved in direct patient care, facilitate discussions on matters related to the workflow and patient and provider burden. These include optimizing how pending issues can be handled more efficiently and derive consensus on how to manage unanticipated situations.

Features of electronic applications clearly play a role in patient engagement and compliance, with integrated communication with care providers and other patients being among the most desirable functionalities, which is included in the Kaiku Health app [35]. The development team considered patients could feel compelled to use the messaging service instead of contacting the medical team via telephone. Given the limited activity period of the nursing triage team, there was considerable risk that some messages would not be addressed in a timely manner, prompting the decision to deactivate this functionality. To accommodate those features in the IePRO model, the flow of communication between patient and providers would need to be revised. The impact on the burden of clinical teams would also need to be considered, as it may result in more frequent prompts to intervene than a system where the decision to initiate contact lies with the provider. Other eHealth interventions have used automated written feedback [35], which could be integrated in this model as well. Ongoing data collection from patient interviews may highlight the strengths and limitations of the application in its current version. Patient feedback will be addressed in future publications.

Data collection concerning the acceptability of the model of care from the provider’s perspective will take place after the trial and will be analyzed and disseminated in a later stage of the project. It is unclear how patients will perceive the novel role of the triage nurse and how it may interfere in their relationship with other providers like the CNS. International guidelines for managing IrAE often require skills, such as prescribing medication and diagnostic tests that most nurses in Switzerland cannot autonomously enact. While close collaboration with physicians in symptom management is essential, the lack of autonomy increases the complexity of the workflow and introduces additional points of failure. Further standardization of practice and continued investment in advanced nursing practice roles may further optimize care delivery and improve the model. Because IrAE management guidelines do not primarily focus on self-management support, some variability in what interventions are put in place by triage nurses is likely. More comprehensive self-management support coverage in those guidelines would empower nurses and patients and further clarify how beneficial outcomes can be achieved [17].

The development of this model benefited from the collaboration with a patient-representative to assess the tools and PROM used in its different components. This triggered deeper discussions with the care team, relating to symptom management and administrative challenges. As patients’ acceptability of the model of care is assessed, we believe future iterations also stand to gain significantly from deeper patient and public involvement.

## Conclusion

The described based model of care provides insight into the complexity of using ePRO data to facilitate potential benefits for both patients and care providers. It attempts to draw a closed feedback loop between patients and providers, to ensure symptoms related to ICI treatments and beyond are monitored and managed by a proactive, prepared provider team.

The IePRO model is not intended as a blueprint for other institutions with that goal. Rather, it is an example of the complexity of such an endeavor, by reworking several components of care in the attempt to generate beneficial outcomes to patients. Under that light, we believe it furthers the discussion around PRO implementation by exposing some of the pragmatic difficulties and compromises that researcher and clinicians may have to manage.

## Supplementary information

ESM 1 (PDF 1529 kb)

ESM 2 (PDF 179 kb)

ESM 3 (PDF 172 kb)

ESM 4 (PDF 186 kb)

## Data Availability

Not applicable.

## Abbreviations

*CCM*: chronic care model
*CDS*: clinical decision support
*CIS*: clinical information systems
*DSD*: delivery system design
*eCCM*: eHealth Enhanced Chronic Care Model
*eHE*: eHealth education
*ePRO*: electronic patient-reported outcomes
*ePROM*: electronic patient-reported outcome measures
*ICI*: immune checkpoint inhibitors
*IrAE*: immune-related adverse events
*MAUQ*: mHealth app usability questionnaire
*PRO*: patient-reported outcomes
*PRO-CTCAE™*: Patient-Reported Outcomes version of the Common Terminology Criteria for Adverse Events
*PROM*: patient-reported outcome measures
*SMS*: self-management support
