# Supplementary material for: Development of an eHealth-enhanced model of care for the monitoring and management of immune-related adverse events in patients treated with immune checkpoint inhibitors
Source: Support Care Cancer. 2023 Jul 22;31(8):484. doi: 10.1007/s00520-023-07934-w (PMC10363070; doi:10.1007/s00520-023-07934-w)
Supplement: Supplementary file 1 — (PDF 1529 kb) [file 520_2023_7934_MOESM1_ESM.pdf]

IEPRO MODE D'EMPLOI

Supplement A : Mobile application guide

Vous êtes inscrit·e dans le Groupe Numérique.

IePRO Information sheet, Version *1 of date 08.03.2022*

A. S'inscrire dans l'application électronique pour remplir les questionnaires

1. Vous recevrez un e-mail avec une invitation à joindre l'application IePRO.

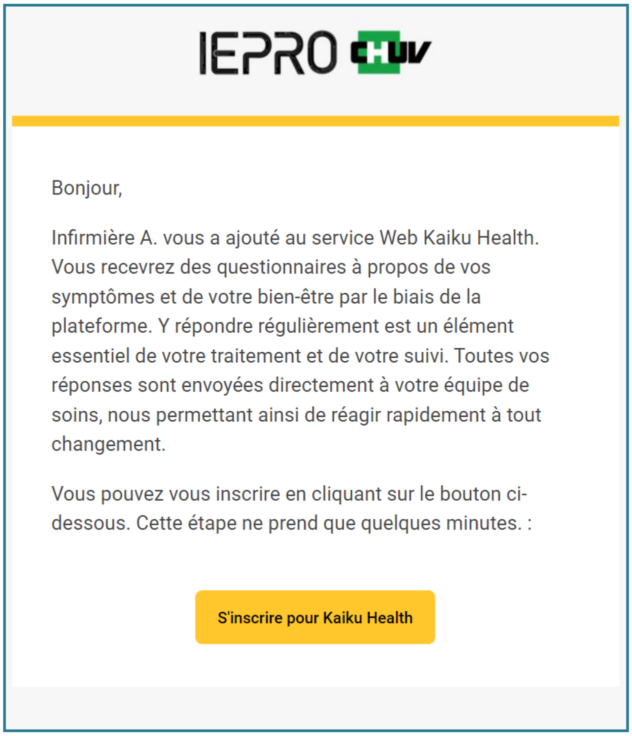

2. Sur la nouvelle page, remplissez toutes les informations nécessaires et cliquez « S'inscrire ».

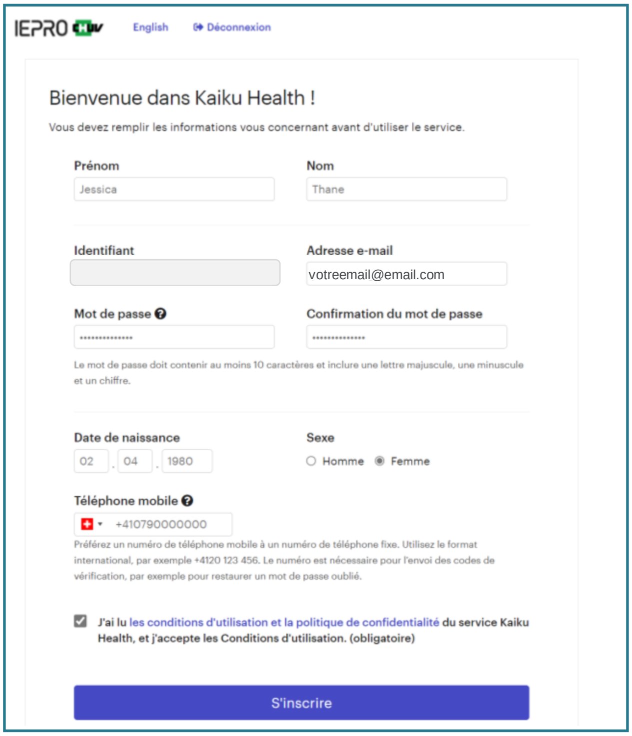

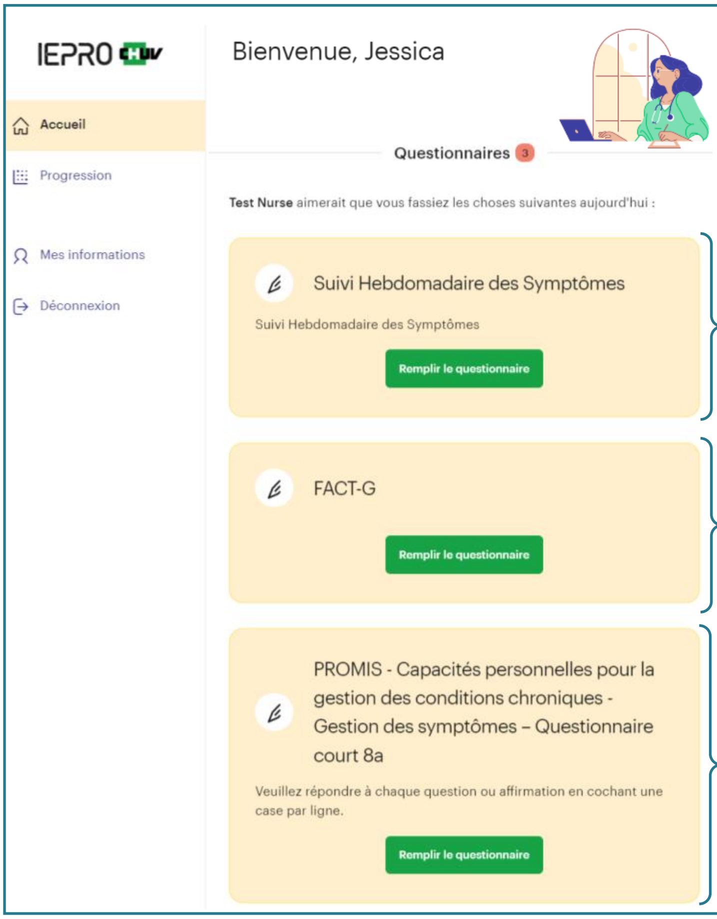

3. Sur la page d'accueil, vous aurez 3 questionnaires à remplir. Après avoir rempli un questionnaire, vous devez retourner à la page d'accueil pour remplir les restants.

a. Le questionnaire hebdomadaire des symptômes à remplir une fois par semaine. Le jour d'après il sera remplacé par le questionnaire journalier.

b. Le questionnaire sur la qualité de vie (FACT-G) à remplir une fois par semaine.

c. Le questionnaire sur l'auto-efficacité pour gérer les symptômes (PROMIS) à remplir une fois par semaine.

MODE D'EMPLOI

Supplement A : Mobile application guide

Vous êtes inscrit·e dans le Groupe Numérique.

IePRO Information sheet, Version *1 of date 08.03.2022*

B. Remplir un questionnaire

1. Une fois que vous ouvrez un questionnaire, vous aurez plusieurs options:

Enregistrer le questionnaire pour le remplir plus tard.

En cliquant ici, vos réponses sont enregistrées mais ne sont pas envoyées à l'équipe soignante. Vous êtes ensuite renvoyé à la page d'accueil.

En rouvrant le questionnaire, vous pouvez continuer à le remplir.

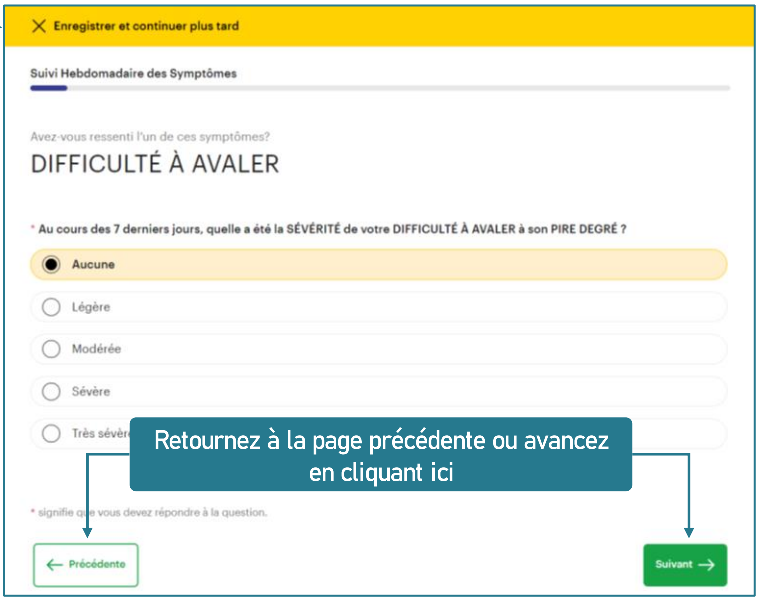

C. Ajouter d'autres symptômes au questionnaire des symptômes hebdomadaire et journalier

Après la dernière question du questionnaire des symptômes, vous avez l'option d'en ajouter d'autres:

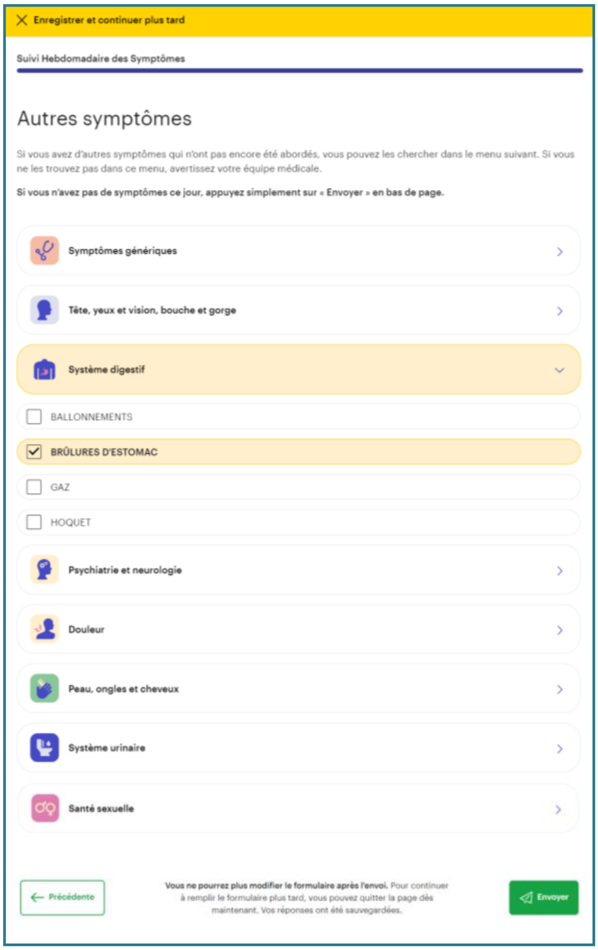

Cliquez sur une catégorie pour dévoiler les options. Choisissez celles que vous voulez.

En cliquant sur « Suivant → », le questionnaire continuera avec les questions concernant le(s) symptôme(s) choisi(s).

Une fois le questionnaire terminé, vous pouvez cliquer sur le bouton Envoyer

Il vous sera alors possible de consulter un résumé de vos réponses.

Si vous n'avez déclaré aucun symptôme, vous rencontrerez cet écran de sélection lorsque vous ouvrirez le questionnaire journalier sur les symptômes.

- Installez l'application Kaiku ou allez sur https://chuv.kaiku.ch pour remplir les questionnaires les jours suivants.
- Si vous avez un problème avec l'application ou si vous avez déclaré un symptôme par erreur, merci de nous le signaler rapidement à : do.id.iepro@chuv.ch

Vous êtes inscrit·e dans le Groupe Soins Standard.

IePRO Information sheet, Version *1 of date 08.03.2022*

A. S'inscrire dans l'application électronique pour remplir les questionnaires

1. Vous recevrez un e-mail avec une invitation à joindre l'application IePRO.

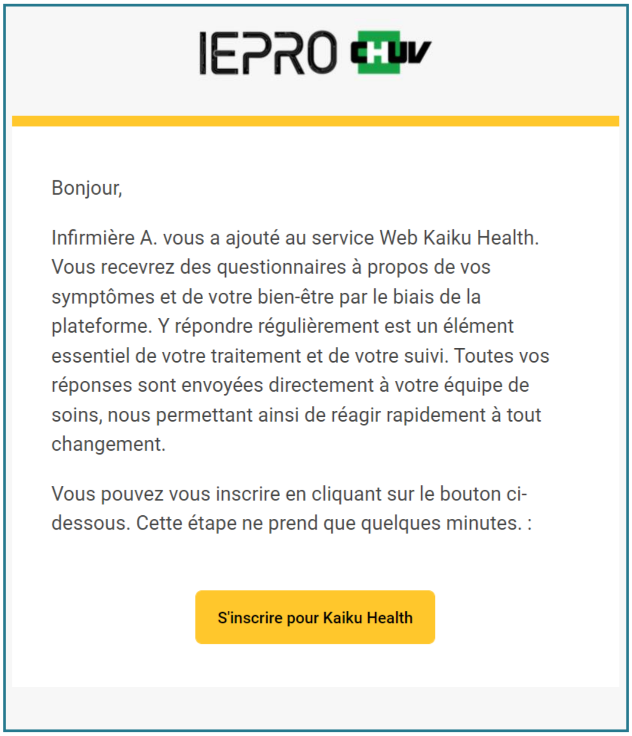

2. Sur la nouvelle page, remplissez toutes les informations nécessaires et cliquez « S'inscrire ».

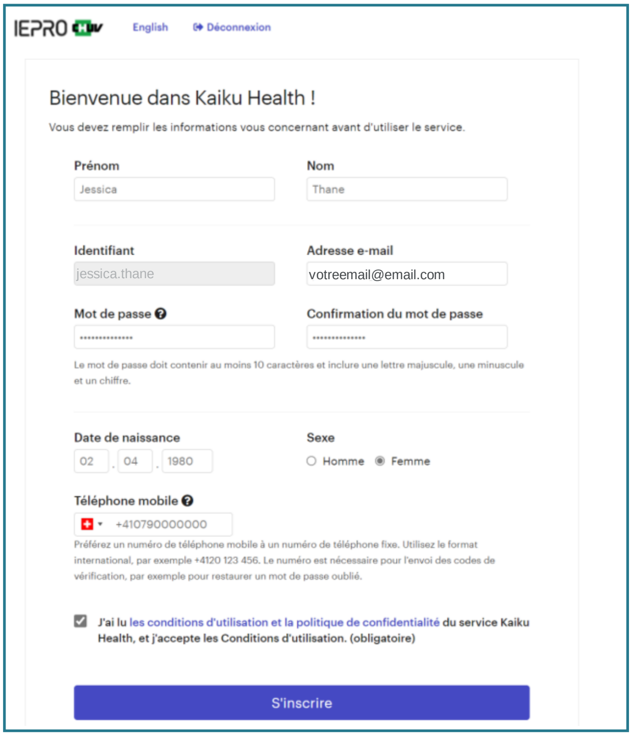

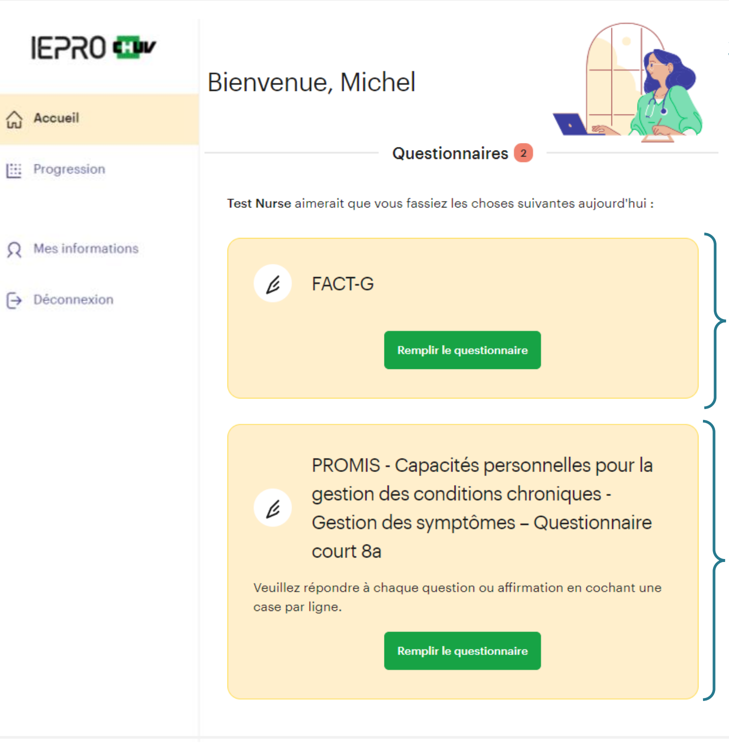

3. Sur la page d'accueil, vous aurez 2 questionnaires à remplir. Après avoir rempli un questionnaire, vous devez retourner à la page d'accueil pour remplir les restants.

a. Le questionnaire sur la qualité de vie (FACT-G) à remplir une fois par semaine.

b. Le questionnaire sur l'auto-efficacité pour gérer les symptômes (PROMIS) à remplir une fois par semaine.

B. Remplir un questionnaire

1. Une fois que vous ouvrez un questionnaire, vous aurez plusieurs options:

Enregistrer le questionnaire pour le remplir plus tard.

En cliquant ici, vos réponses sont enregistrées mais ne sont pas envoyées à l'équipe soignante. Vous êtes ensuite renvoyé à la page d'accueil.

En rouvrant le questionnaire, vous pouvez continuer à le remplir.

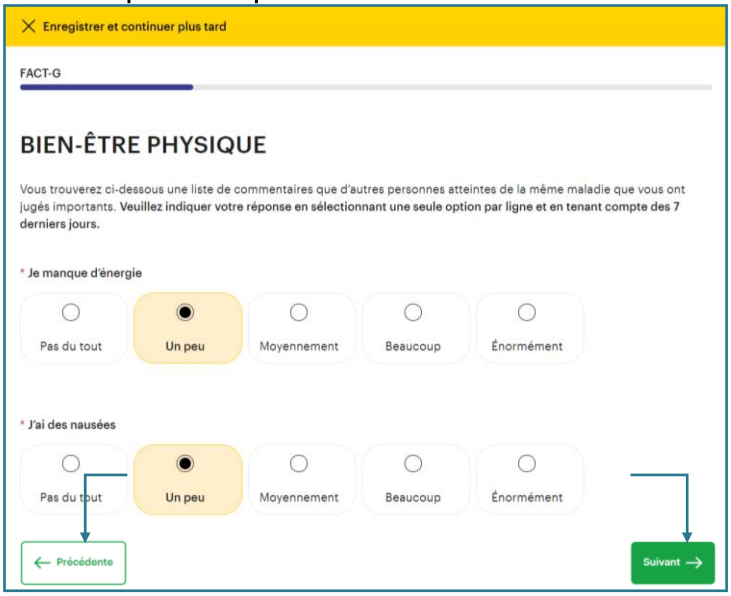

Une fois le questionnaire terminé, vous pouvez cliquer sur le bouton 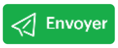

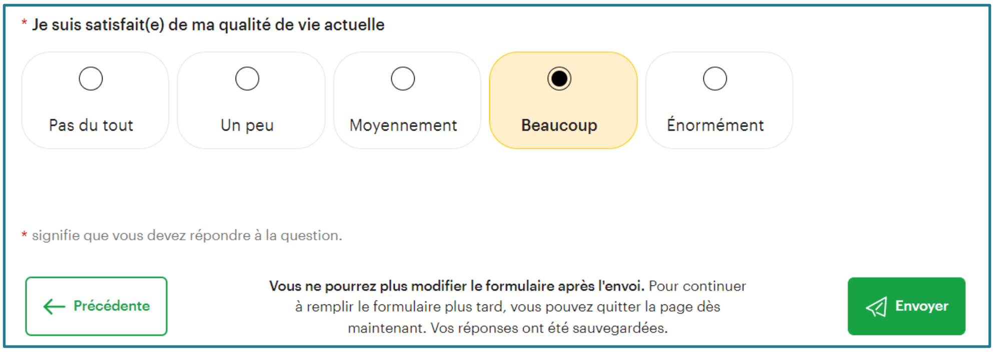

Vous retournerez ensuite à l'écran d'accueil. Un message de confirmation de l'envoi du questionnaire s'affichera:

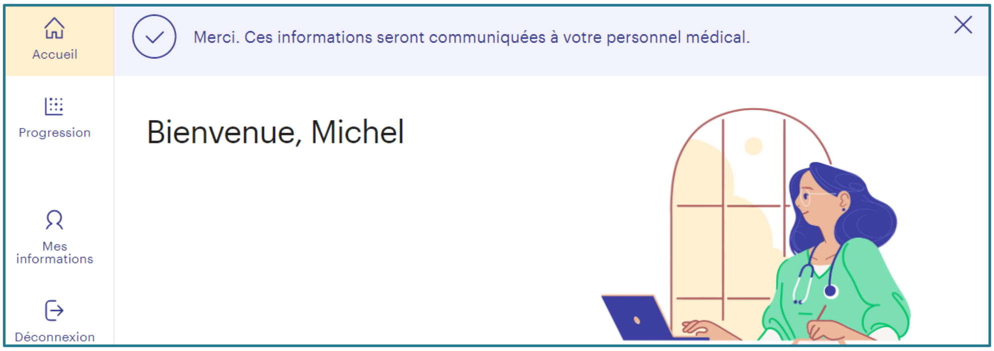

Installez l'application Kaiku ou allez sur https://chuv.kaiku.ch
pour remplir les questionnaires les jours suivants.

◆

Si vous avez un problème avec l'application ou si vous avez déclaré un symptôme par erreur, merci de nous le signaler rapidement à : do.id.iepro@chuv.ch
