# Supplementary material for: Development of an eHealth-enhanced model of care for the monitoring and management of immune-related adverse events in patients treated with immune checkpoint inhibitors
Source: Support Care Cancer. 2023 Jul 22;31(8):484. doi: 10.1007/s00520-023-07934-w (PMC10363070; doi:10.1007/s00520-023-07934-w)
Supplement: Supplementary file 2 — (PDF 179 kb) [file 520_2023_7934_MOESM2_ESM.pdf]

Supplement B : Mobile application usability interview guide

For: Development of an ehealth-enhanced model of care for the monitoring and management of immune-related adverse events in patients treated with immune-checkpoint inhibitors (2022)

Based on the mHealth App Usability Questionnaire (MAUQ) by Zhou L et al (2019) [1]

The original French version of this semi-structured interview guide was translated to English

**When interviews will be conducted:**	Within 2 weeks after the trial, the same day of a scheduled follow-up consultation.
**Where interviews will be conducted:**	On site, in a closed, private room.
**Who will conduct the interviews**	Study investigators

THEMES AND GUIDING QUESTIONS

WELCOME & INTRODUCTION

- Thank you for your participation - we are all very grateful for your time and cooperation.
- We would like to record these discussions so that we can listen to them again if necessary, to ensure that we don't miss any of the ideas or issues raised. The details of these discussions will not be shared with anyone else outside this study; your name will be kept confidential and no one else will know what was said during our conversations. Please feel free to express your opinions openly in order to get the best possible representation of reality. We are particularly interested in areas that can be improved.
- If you are not comfortable with these elements you are not obligated to participate. Are you willing to participate in this interview?
- This is an open space to discuss your experience.

**1. How do you feel about the usability of this application?**	
Follow-up questions:	How do you like the navigation of the application?
	How was it to find what you were looking for in the application?
	Can you give examples of easy or difficult things about it?
**2. How did you find the information provided by the application?**	
Follow-up questions:	What do you think about the clarity of the information provided by the application?
	Did the information seem relevant to you?
**3. How do you feel about the time it took you to use the application?**	
Follow-up questions:	How long do you think it took you to use it per day?
**4. Have you used this application in public? Did you feel comfortable doing so?**	
Follow-up questions:	If not, can you explain why?
**5. How has this application affected your interactions with healthcare professionals?**	

Follow-up questions:	Has it influenced your interactions with healthcare professionals (physicians, nurses,...)?
	Can you give an example?
	Can you explain why?
**6. Was there a moment when you questioned if your answers to the questionnaires had reached the nurse?**	
Relance	If so, when and why?
Relance	If not, why ?
**7. Overall, what did you think of the application? Did it meet your expectations?**	
Follow-up questions:	Did the application meet your expectations? (If so/not,) Can you explain why?
	Did you like it or dislike it? Can you point out any reasons for this?
8. **Between 0 and 10, how would you rate your overall experience with the application (0 - Poor, 10 - Excellent)?**	
9. **Would you recommend it to other people with cancer?**	
Follow-up questions:	If so, why ?
	If not, why not ?
**10. Do you have any other comments or observations about the application that you would like to share?**	

[1] Zhou L, Bao J, Setiawan IMA, Saptono A, Parmanto B. The mHealth App Usability Questionnaire (MAUQ): Development and Validation Study. JMIR Mhealth Uhealth 2019;7:e11500. https://doi.org/10.2196/11500.
