# Supplementary material for: Development of an eHealth-enhanced model of care for the monitoring and management of immune-related adverse events in patients treated with immune checkpoint inhibitors
Source: Support Care Cancer. 2023 Jul 22;31(8):484. doi: 10.1007/s00520-023-07934-w (PMC10363070; doi:10.1007/s00520-023-07934-w)
Supplement: Supplementary file 3 — (PDF 172 kb) [file 520_2023_7934_MOESM3_ESM.pdf]

Supplement C : Semi-structured interview for patients to assess the acceptability of the IePRO model of care

For: Development of an ehealth-enhanced model of care for the monitoring and management of immune-related adverse events in patients treated with immune-checkpoint inhibitors (2022)

Based on Acceptability of healthcare interventions: an overview of reviews and development of a theoretical framework by Sekhon et al, 2017 [1]

The original French version of this semi-structured interview guide was translated to English

**When interviews will be conducted:**	Within 2 weeks after the trial, the same day of a scheduled follow-up consultation.
**Where interviews will be conducted:**	On site, in a closed, private room.
**Who will conduct the interviews**	Study investigators

THEMES AND GUIDING QUESTIONS

WELCOME & INTRODUCTION

- Thank you for your participation - we are all very grateful for your time and cooperation.
- We would like to record these discussions so that we can listen to them again if necessary, to ensure that we don't miss any of the ideas or issues raised. The details of these discussions will not be shared with anyone else outside this study; your name will be kept confidential and no one else will know what was said during our conversations. Please feel free to express your opinions openly in order to get the best possible representation of reality. We are particularly interested in areas that can be improved.
- If you are not comfortable with these elements you are not obligated to participate. Are you willing to participate in this interview?
- This is an open space to discuss your experience.

**1. When you think about this model of care, how do you feel?**	
Follow-up questions:	Do you feel safe or unsafe, relieved or frustrated about the care?
**2. What were your expectations of this model of care at the beginning of this study? To what extent have they been met?**	
**3. How did you feel when you received a call from the nurse?**	
Follow-up questions:	What was your experience with the nurse interactions over the phone?
**4. Do you feel like you needed to make a significant effort to participate in this type of care?**	
Follow-up questions:	If so, can you give examples of this effort?<br>If not, why?
**5. Do you feel that this model of care has impacted the way you manage your symptoms?**	
Follow-up questions:	Do you feel that this way of providing care has played a role in how you manage your symptoms?

	Did this type of care work for you or not work for you in managing your symptoms?
	Do you feel that the electronic application played a role in how you manage your symptoms?

[1] Sekhon M, Cartwright M, Francis JJ. Acceptability of healthcare interventions: an overview of reviews and development of a theoretical framework. BMC Health Services Research 2017;17. https://doi.org/10.1186/s12913-017-2031-8.
