# Supplementary material for: Development of an eHealth-enhanced model of care for the monitoring and management of immune-related adverse events in patients treated with immune checkpoint inhibitors
Source: Support Care Cancer. 2023 Jul 22;31(8):484. doi: 10.1007/s00520-023-07934-w (PMC10363070; doi:10.1007/s00520-023-07934-w)
Supplement: Supplementary file 4 — (PDF 186 kb) [file 520_2023_7934_MOESM4_ESM.pdf]

Supplement D : Semi-structured interview for nurses to assess the implementation of the IePRO model of care

For: Development of an ehealth-enhanced model of care for the monitoring and management of immune-related adverse events in patients treated with immune-checkpoint inhibitors (2022)

Based on the Consolidated Framework for Implementation Research (CFIR) guide: *https://cfirguide.org/guide/app/guide.html*

The original French version of this semi-structured interview guide was translated to English

**When interviews will be conducted:**	Within 6 weeks after the trial, individually.
**Where interviews will be conducted:**	On site, in a closed, private room.
**Who will conduct the interviews**	Study investigator

Outer Setting

Patient Needs & Resources

1. To what extent were the needs and preferences of the patients considered when deciding to implement the ePRO-based model of care?
   - Can you describe specific examples?
   - Will the ePRO-based model of care be altered to meet their needs and preferences?
2. How well do you think the ePRO-based model of care will meet the needs of patients?
   - In what ways will the model of care meet their needs? E.g. improved access to services? Reduced wait times? Help with self-management? Reduced travel time and expense?
3. How do you think the patients will respond to the ePRO-based model of care?
4. What barriers will the patients face to participating in the ePRO-based model of care?
5. Have you heard stories about the experiences of participants with the intervention?
   - Can you describe a specific event?

Inner Setting

Structural Characteristics

1. What kinds of infrastructure changes will be needed to accommodate the ePRO-based model of care?
   - Changes in scope of practice? Changes in formal policies? Changes in information systems or electronic records systems? Other?
   - What kind of approvals will be needed? Who will need to be involved?
   - Can you describe the process that will be needed to make these changes?

Characteristics of Individuals

Knowledge & Beliefs about the Intervention

1. How do you feel about the ePRO-based model of care being used in your setting?
   - How do you feel about the plan to implement the intervention in your setting?

- Do you have any feelings of anticipation? Stress? Enthusiasm? Why?

Self-efficacy

1. How confident do you think your colleagues feel about using the intervention?
    - What gives them that level of confidence (or lack of confidence)?

Process

Executing

1. Has the intervention been applied during the study according to the plan?
    - [If Yes] Can you describe this?
    - [If No] Why not?

Other important issues related to the ePRO-based model of care

1. Is there any other theme or issue that you would like to share regarding your experiences with the ePRO-based model of care?

[1] Sekhon M, Cartwright M, Francis JJ. Acceptability of healthcare interventions: an overview of reviews and development of a theoretical framework. BMC Health Services Research 2017;17. https://doi.org/10.1186/s12913-017-2031-8.
